# *TRPA1* gene polymorphisms and childhood asthma

**DOI:** 10.1111/pai.12673

**Published:** 2016-12-08

**Authors:** Valentina Gallo, F. Nicole Dijk, John W. Holloway, Susan M. Ring, Gerard H. Koppelman, Dirkje S. Postma, David P. Strachan, Raquel Granell, Johan C. de Jongste, Vincent W. V. Jaddoe, Herman T. den Dekker, Liesbeth Duijts, A. John Henderson, Seif O. Shaheen

**Affiliations:** ^1^ Centre for Primary Care and Public HealthBarts and The London School of Medicine and DentistryLondonUK; ^2^ Department of Pediatric Pulmonology and Pediatric AllergologyBeatrix Children's HospitalGroningen Research Institute for Asthma and COPDUniversity Medical Center GroningenUniversity of GroningenGroningenThe Netherlands; ^3^ Human Development and HealthFaculty of MedicineUniversity of SouthamptonSouthamptonUK; ^4^ School of Social and Community MedicineUniversity of BristolBristolUK; ^5^ MRC Integrative Epidemiology Unit at the University of BristolUniversity of BristolBristolUK; ^6^ Department of PulmonologyGroningen Research Institute for Asthma and COPDUniversity Medical Center GroningenUniversity of GroningenGroningenThe Netherlands; ^7^ St George'sUniversity of LondonLondonUK; ^8^ Division of Respiratory MedicineDepartment of PediatricsErasmus University Medical CenterRotterdamThe Netherlands; ^9^ The Generation R Study GroupErasmus University Medical CenterRotterdamThe Netherlands; ^10^ Department of of EpidemiologyErasmus University Medical CenterRotterdamThe Netherlands; ^11^ Department of PediatricsErasmus University Medical CenterRotterdamThe Netherlands; ^12^ Division of NeonatologyDepartment of PediatricsErasmus University Medical CenterRotterdamThe Netherlands

**Keywords:** Avon Longitudinal Study of Parents and Children, asthma, birth cohort, Generation R, gene–environment interaction, genotype, paracetamol, Prevention and Incidence of Asthma and Mite Allergy, prenatal exposure, transient receptor potential ankyrin‐1

## Abstract

**Background:**

Animal data have suggested that the transient receptor potential ankyrin‐1 (TRPA1) ion channel plays a key role in promoting airway inflammation in asthma and may mediate effects of paracetamol on asthma, yet confirmatory human data are lacking. To study associations of *TRPA1* gene variants with childhood asthma and total IgE concentration, and interactions between *TRPA1* and prenatal paracetamol exposure on these outcomes.

**Methods:**

We analysed associations between 31 *TRPA1* single nucleotide polymorphisms (SNPs) and current doctor‐diagnosed asthma and total IgE concentration at 7.5 years in the Avon Longitudinal Study of Parents and Children (ALSPAC) birth cohort. We sought to confirm the most significant associations with comparable outcomes in the Prevention and Incidence of Asthma and Mite Allergy (PIAMA) and Generation R birth cohorts. In ALSPAC, we explored interactions with prenatal paracetamol exposure.

**Results:**

In ALSPAC, there was strong evidence for association between six SNPs and asthma: rs959974 and rs1384001 (per‐allele odds ratio for both: 1.30 (95% CI: 1.15–1.47), p = 0.00001), rs7010969 (OR 1.28 (1.13–1.46), p = 0.00004), rs3735945 (OR 1.30 (1.09–1.55), p = 0.003), rs920829 (OR 1.30 (1.09–1.54), p = 0.004) and rs4738202 (OR 1.22 (1.07–1.39), p = 0.004). In a meta‐analysis across the three cohorts, the pooled effect estimates confirmed that all six SNPs were significantly associated with asthma. In ALSPAC,*TRPA1* associations with asthma were not modified by prenatal paracetamol, although associations with IgE concentration were.

**Conclusion:**

This study suggests that TRPA1 may play a role in the development of childhood asthma. (249 words)

**Abbreviations**
TRPA1: transient receptor potential ankyrin‐1
ALSPAC: Avon Longitudinal Study of Parents and Children
PIAMA: Prevention and Incidence of Asthma and Mite Allergy
SNP: single nucleotide polymorphism
PAF: population attributable fraction
LD: linkage disequilibrium

## Introduction

The transient receptor potential ankyrin‐1 (TRPA1) ion channel is expressed on peripheral endings of primary afferent neurons and is a highly conserved sensor of noxious reactive electrophiles; these form covalent adducts with the receptor to activate the neurons 1. In particular, TRPA1 is a major oxidant sensor in the airways 2, sensing exogenous airborne irritants as well as endogenous by‐products of oxidative stress 3. In keeping with this function, the TRPA1 receptor is thought to play a key role in the cough reflex 4 and in promoting airway inflammation in asthma 3, 5. Experiments using knockout mice and TRPA1 antagonists have shown that TRPA1 plays a critical role in allergic and non‐allergic neurogenic airway inflammation and hyper‐reactivity 6, 7. However, evidence implicating TRPA1 in asthma in humans is lacking.

Following our initial discovery of an association between frequent paracetamol (acetaminophen) use and asthma in adults 8, we and others have reported that maternal use of paracetamol in pregnancy was associated with an increased risk of childhood asthma, wheezing and elevated total IgE concentration 9. Nassini et al. 10 subsequently showed in a rodent model that systemic administration of therapeutic doses of paracetamol led to generation of its electrophilic and reactive metabolite in the lung which, in turn, caused neurogenic airway inflammation through activation of TRPA1; they proposed that this mechanism might explain the epidemiological link between paracetamol exposure and asthma in humans.

In a population‐based birth cohort, we investigated whether *TRPA1* (8q13) gene variants are associated with childhood asthma and IgE concentration, and whether these associations were modified by prenatal exposure to paracetamol. We also sought to obtain confirmatory evidence for the most significant SNP associations in the Prevention and Incidence of Asthma and Mite allergy (PIAMA) and Generation R birth cohorts.

## Methods

### Avon Longitudinal Study of Parents and Children

#### Subjects

The Avon Longitudinal Study of Parents and Children (ALSPAC) is a population‐based birth cohort that recruited 14,541 predominantly white pregnant women resident in Avon, UK, with expected dates of delivery 1st April 1991–31st December 1992. Of these pregnancies, there were 14,062 live births and 13,988 children alive at 1 year of age. The cohort has been followed since birth with annual questionnaires and, since age 7 years, with objective measures in research clinics. The study protocol has been described previously 11, 12 (further information at: http://www.alspac.bris.ac.uk). Ethics approval was obtained from the ALSPAC Ethics and Law Committee (IRB 00003312) and the Local Research Ethics Committees.

#### Outcomes

When the children were 7.5 years old, mothers were asked: ‘Has your child had any of the following in the past 12 months: wheezing; asthma?’ Children were defined as having current doctor‐diagnosed asthma (primary outcome) if mothers responded positively to the question ‘Has a doctor ever actually said that your study child has asthma?’ and positively to one or both of the questions on wheezing and asthma in the past 12 months.

Serum total IgE concentration (kU/l) was measured by fluoroimmunoassay using the Pharmacia UNICAP system (Pharmacia & Upjohn Diagnostics AB, Uppsala, Sweden) at 7 years.

#### Prenatal paracetamol exposure

Mothers were asked at 18–20 weeks how often they had taken paracetamol (‘not at all, sometimes, most days, every day’) during their pregnancy. At 32 weeks, they were asked the same question about use in the previous 3 months. Hence, we defined use of paracetamol (Yes/No) in early (<18–20 weeks) and late (20–32 weeks) pregnancy.

#### Genotyping and selection of *TRPA1* SNPs

DNA samples were extracted from lymphoblastoid cell lines, cord blood or venous blood collected at 7 years of age, with a small number extracted from venous blood collected at 43–61 months. A total of 9912 subjects were genotyped at 500,527 SNPs using the Illumina HumanHap550 quad genomewide SNP genotyping platform. After applying rigorous exclusion criteria, genotype data were available for 8365 unrelated individuals (see Online Supplement for further details).

We identified 29 SNPs in *TRPA1* (8q13) which had been included in a genetic association study of cough 13. The participating cohorts in that study were part of a large European GWAS of asthma (the GABRIEL consortium) 14. All SNPs within the gene region had been selected, allowing capture of the majority of common haplotype variations of the gene 13, 14. In addition, we identified 11 SNPs (four of which had already been selected) associated with various pain phenotypes 15, 16, 17 and with menthol preference in smokers 18. Of the 36 potential SNPs, five had not been typed or could not be imputed, leaving 31 SNPs to be analysed. Of these SNPs, 21 were genotyped and 10 were imputed. Where genotyped data were missing, these were replaced by imputed data if possible (see Online Table S1 and Supplement for further details).

#### Statistical analysis of ALSPAC data

Although the GWAS data set only included individuals of European ancestry, we excluded mother–child pairs from all analyses if the mother's reported ethnicity was non‐white or unknown (14.1% of the cohort) to further reduce potential confounding by population substructure. We used logistic regression to analyse relations of child *TRPA1* genotype with asthma, and linear regression to analyse associations with log‐transformed total IgE concentration. All analyses were carried out using Stata (version 10.1). Univariate gene main effects were evaluated as continuous per‐allele effects and using between genotype comparisons. We used Haploview 19 to compute linkage disequilibrium (LD) statistics for the 31 *TRPA1* SNPs of interest. The population attributable fraction (PAF) was calculated using the formula: PAF = 1‐PUF, where PUF is the population unattributable fraction 20. We used the Nyholt approach 21 updated by Li and Ji 22 to estimate the effective number of independent marker loci in our data (12.8 of 31) and the threshold required to keep type I error rate at 5% after adjusting for multiple testing (p value=0.05/12.8 = 0.004).

### PIAMA and Generation R (Netherlands)

The Prevention and Incidence of Asthma and Mite Allergy (PIAMA) birth cohort is a multicentre study that selected 4146 pregnant women in the Netherlands in 1996/1997 23, 24. The Generation R Study is a population‐based prospective cohort study of pregnant women and their children in Rotterdam 25, 26. All children were born between April 2002 and January 2006, and currently followed until young adulthood. Current doctor‐diagnosed asthma at 8 years and at 6 years was defined in PIAMA and Generation R, respectively (see Online Supplement for further details).

We analysed the associations between *TRPA1* (for SNPs most significantly associated with asthma in ALSPAC) and asthma separately in PIAMA and Generation R, and then undertook a meta‐analysis across the three cohorts, using a fixed‐effects model.

### Other European asthma studies

In other European studies included in the GABRIEL study 14, we explored associations between doctor‐diagnosed asthma ‘ever’ (of childhood‐onset) and the *TRPA1* SNPs most significantly associated with asthma in ALSPAC. We carried out these subsidiary analyses using publicly available data from GABRIEL and meta‐analysed the data using a fixed‐effects model.

## Results

In ALSPAC, information on current doctor‐diagnosed asthma at age 7.5 years was obtained for 7221 children. After excluding non‐white mother–child pairs, and applying quality criteria to imputed genotype data, *TRPA1* genotype data were available for 6901 children, generating a final sample of 5141 white children with complete data on asthma and genotype, of whom 614 (11.9%) children had current doctor‐diagnosed asthma at age 7.5 years. A total of 53.9% and 42.3% of children were exposed to paracetamol *in utero* during early and late pregnancy, respectively. Data on total IgE concentration and genotype were available for 3834 children.

*TRPA1* genotype data are summarized in Table S1. *TRPA1* genotype frequencies did not deviate from Hardy–Weinberg equilibrium for the 31 SNPs of interest (p > 0.05). In PIAMA, information on current doctor‐diagnosed asthma at age 8 years was obtained for 3253 children, and *TRPA1* genotype data were available for 1968 children, generating a final sample of 1877 white children with data on asthma and genotype, of whom 89 (4.7%) had current doctor‐diagnosed asthma at age 8 years. In Generation R, data on *TRPA1* genotype and current doctor‐diagnosed asthma at age 6 years were available for 2073 children, after excluding twins and restricting to Caucasians only, based on genetic ancestry. Of these, 64 children (3.1%) had current doctor‐diagnosed asthma.

### Gene main effects in ALSPAC

Table 1 shows the per‐allele associations between *TRPA1* genotypes and asthma in ALSPAC. Of the 31 SNPs tested, 13 were associated with asthma (p < 0.05). The six SNPs (five genotyped, one imputed) that were most significantly associated with asthma (p < 0.005) were as follows: rs959974 and rs1384001 (per‐allele odds ratio for both SNPs: 1.30 (95% CI: 1.15–1.47), p = 0.00001), rs7010969 (OR 1.28 (1.13–1.46), p = 0.00004), rs3735945 (OR 1.30 (1.09–1.55), p = 0.003), rs920829 (OR 1.30 (1.09–1.54), p = 0.004) and rs4738202 (OR 1.22 (1.07–1.39), p = 0.004). Adjustment for multiple testing suggested that associations with these six SNPs (and especially the first four) were unlikely to have arisen by chance (adjusted p value threshold 0.004). With a more rigorous p value threshold of 0.001, evidence against the null hypothesis was still very strong for 3 SNPs.

**Table 1:** Per‐allele associations between child *TRPA1* SNPs and current doctor‐diagnosed asthma at 7.5 years in ALSPAC

SNP	Position	Doctor‐diagnosed asthma at 7 years		
		N	OR (95% CI)	p value
rs12540984	72927920	5110	1.00 (0.84–1.18)	0.985
rs4738201	72930711	5140	1.16 (1.03–1.31)	0.013
rs6996723	72933632	5141	0.88 (0.75–1.04)	0.137
rs7827617	72934032	5141	1.21 (1.04–1.41)	0.013
rs959974	72935839	5141	1.30 (1.15–1.47)	0.00001
rs959976	72936145	5141	1.22 (1.05–1.42)	0.008
rs1384001	72936237	5141	1.30 (1.15–1.47)	0.00001
rs13279503	72939626	5116	1.08 (0.95–1.22)	0.222
rs4738202	72940861	5141	1.22 (1.07–1.39)	0.004
rs13280644	72948588	5141	0.82 (0.66–1.02)	0.075
rs13249568	72949209	5141	0.95 (0.83–1.09)	0.468
rs10504523	72951490	5141	0.95 (0.83–1.09)	0.484
rs1025926	72953158	5141	1.14 (1.00–1.30)	0.055
rs10504524	72955891	5141	0.95 (0.83–1.09)	0.479
rs13255063	72959535	5140	0.95 (0.83–1.09)	0.476
rs1025927	72963135	5138	0.82 (0.66–1.01)	0.067
rs1025928	72963258	5141	0.94 (0.83–1.07)	0.344
rs10504525	72965123	5141	1.06 (0.90–1.25)	0.494
rs3735942	72965973	5141	1.11 (0.98–1.26)	0.097
rs3735943	72966002	5141	0.88 (0.78–0.99)	0.040
rs10504526	72966552	5141	1.13 (1.01–1.28)	0.041
rs12548486	72971527	5138	1.11 (0.98–1.26)	0.102
rs10109581	72974329	5141	1.19 (1.05–1.36)	0.009
rs3735945	72974806	5141	1.30 (1.09–1.55)	0.003
rs920829	72977703	5136	1.30 (1.09–1.54)	0.004
rs1443952	72980652	5141	1.11 (0.98–1.25)	0.116
rs7010969	72982365	5141	1.28 (1.13–1.46)	0.00004
rs7011431	72982398	5141	1.20 (1.05–1.36)	0.008
rs4738206	72986348	5141	1.10 (0.97–1.25)	0.120
rs2278655	72987277	5038	1.01 (0.79–1.28)	0.964
rs13268757	72987638	5097	1.06 (0.89–1.25)	0.528

Additional effect estimates using between genotype comparisons for these six SNPs in relation to asthma are shown in Table 2. This shows that, for four of these SNPs, children who were homozygous for the risk allele were approximately 70% more likely to have asthma than children who were homozygous for the non‐risk allele. Of the 31 SNPs tested, only three (rs959974, rs1384001, rs4738202) were nominally associated with total IgE concentration (p < 0.05) (Table S2).

**Table 2:** Associations between the six most significantly associated *TRPA1* SNPs in ALSPAC and current doctor‐diagnosed asthma at 7–8 years in ALSPAC and PIAMA, and current doctor‐diagnosed asthma at 6 years in Generation R

SNP	Alleles	ALSPAC			PIAMA			GENERATION R		
		N	OR	p‐value	N	OR	p‐value	N	OR	p‐value
rs959974^a^	G/G	1401	1.00		512	1.00		555	1.00	
	G/T	2615	1.33 (1.07–1.65)	0.009	932	1.36 (0.77–2.38)	0.28	1054	1.15 (0.61, 2.14)	0.67
	T/T	1125	1.69 (1.32–2.16)	0.00001	433	1.82 (0.99–3.36)	0.053	464	1.39 (0.68, 2.82)	0.37
	Per allele		1.30 (1.15–1.47)	0.00001		1.35 (1.00–1.83)	0.052		1.18 (0.83, 1.68)	0.37
rs1384001^b^	C/C	1400	1.00		512	1.00		555	1.00	
	A/C	2616	1.33 (1.07–1.65)	0.009	933	1.36 (0.78–2.38)	0.28	1054	1.15 (0.61, 2.14)	0.67
	A/A	1125	1.69 (1.32–2.15)	0.00001	432	1.83 (0.99–3.37)	0.053	464	1.39 (0.68, 2.82)	0.37
	Per allele		1.30 (1.15–1.47)	0.00001		1.35 (1.00–1.83)	0.051		1.18 (0.83, 1.68)	0.37
rs4738202^a^	A/A	483	1.00		150	1.00		179	1.00	
	A/G	2233	1.45 (1.02–2.05)	0.038	816	1.54 (0.54–4.41)	0.42	880	0.71 (0.30, 1.68)	0.44
	G/G	2425	1.66 (1.18–2.34)	0.004	911	2.21 (0.79–6.20)	0.13	1,014	0.78 (0.34, 1.81)	0.57
	Per allele		1.22 (1.07–1.39)	0.004		1.45 (1.01–2.09)	0.042		0.96 (0.65, 1.41)	0.83
rs7010969^b^	A/A	827	1.00		299	1.00		324	1.00	
	A/C	2477	1.43 (1.09–1.89)	0.010	920	1.09 (0.56–2.10)	0.80	1005	1.08 (0.51, 2.32)	0.84
	C/C	1837	1.74 (1.31–2.29)	0.00005	658	1.42 (0.73–2.77)	0.30	744	1.20 (0.55, 2.62)	0.65
	Per allele		1.28 (1.13–1.46	0.00004		1.23 (0.89–1.68)	0.21		1.10 (0.76, 1.59)	0.61
rs3735945^b^	C/C	4067	1.00		1519	1.00		1621	1.00	
	C/T	1005	1.38 (1.13–1.68)	0.002	338	1.15 (0.67–1.95)	0.61	428	1.40 (0.80, 2.47)	0.24
	T/T	69	1.19 (0.59–2.41)	0.633	20	0.00 (0.00 to –)^c^	0.99	24	0.00 (0.00 to –)^c^	0.99
	Per allele		1.30 (1.09–1.55)	0.003		1.01 (0.61–1.65)	0.98		1.21 (0.71, 2.04)	0.48
rs920829^d^	C/C	4066	1.00		1519	1.00		1621	1.00	
	C/T	1001	1.37 (1.12–1.68)	0.002	338	1.15 (0.67–1.95)	0.61	428	1.40 (0.80, 2.47)	0.24
	T/T	69	1.19 (059–2.41)	0.634	20	0.00 (0.00 to –)^c^	0.99	24	0.00 (0.00 to –)^c^	0.99
	Per allele		1.30 (1.09–1.54)	0.004		1.01 (0.61–1.65)	0.98		1.21 (0.71, 2.04)	0.48

a Genotyped in ALSPAC and in PIAMA, and imputed in Generation R.

b Genotyped in ALSPAC, and imputed in PIAMA and Generation R.

c No asthma cases in minor allele homozygote group in PIAMA and Generation R.

d Imputed in ALSPAC and in PIAMA, and genotyped in Generation R.

Fig. S1 in the online supplement shows LD (r^2^) between the 31 *TRPA1* SNPs; 29 of those SNPs are located in four LD blocks. Of the six SNPs most significantly associated with asthma, two (rs959974 and rs1384001) were in one block, rs4738202 was in another block, and rs7010969, rs3735945 and rs920829 were in a third block. We chose three of the most significantly associated SNPs from different LD blocks (rs959974, rs7010969 and rs4738202) to separately estimate the proportion of asthma in the population attributable to *TRPA1* genotype (PAF). The PAF estimates were, respectively, 21.7% (95% CI: 9.6–32.2; p = 0.001), 29.1% (12.5–42.6; p = 0.001) and 30.7% (7.7–47.9; p = 0.012).

### Gene main effects in PIAMA and Generation R and meta‐analysis

**Figure 1:**
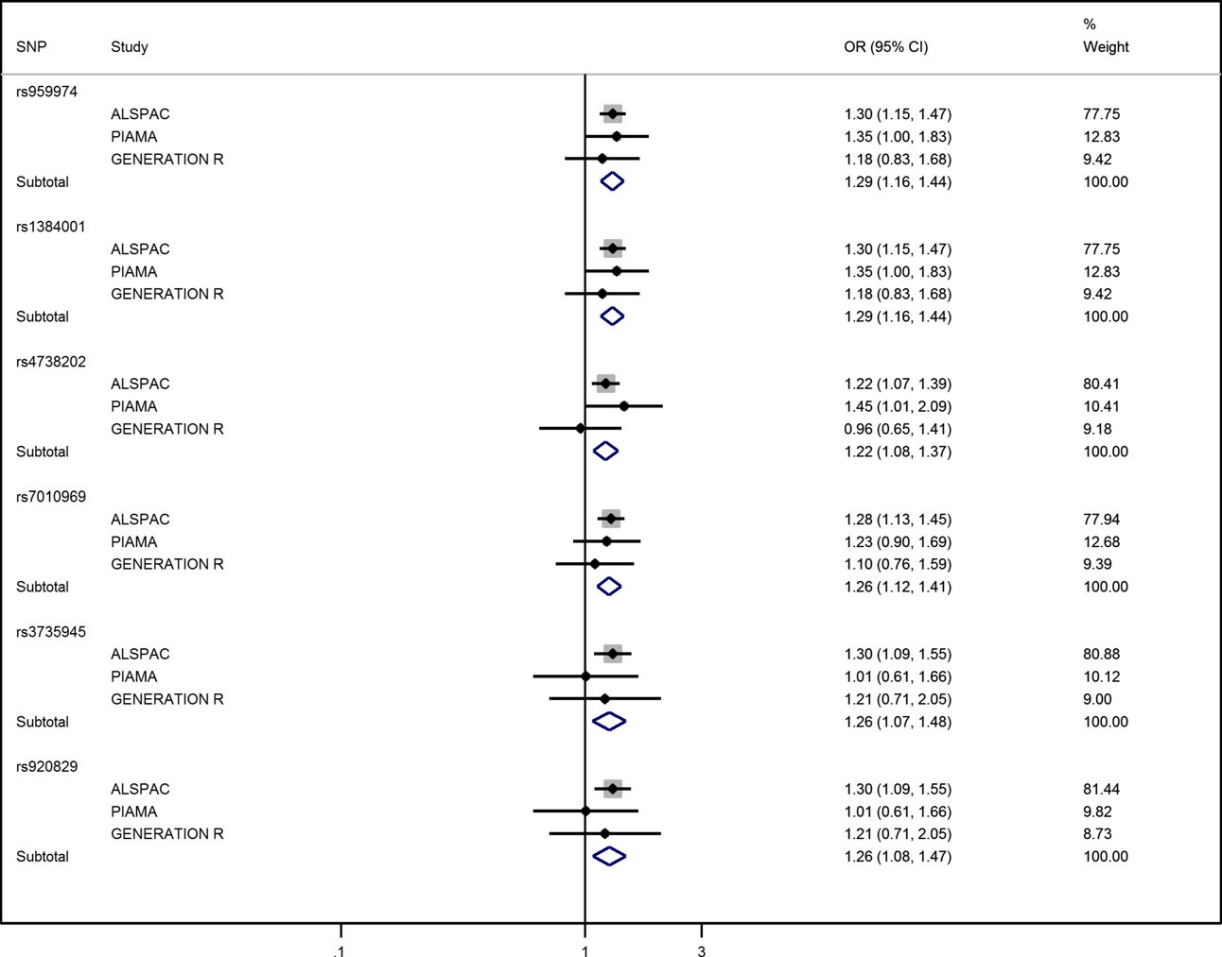
Forest plots showing meta‐analysis of the per‐allele associations between the six *TRPA1* SNPs most significantly associated with asthma in ALSPAC and current asthma in ALSPAC, PIAMA and Generation R.

Table 2 also shows the associations between the six SNPs most significantly associated with asthma in ALSPAC and asthma in the PIAMA and Generation R cohorts. In PIAMA, there was some evidence for association (p ≤ 0.05) with asthma for the three SNPs most significantly associated with asthma in ALSPAC, with effect estimates that were larger than those in ALSPAC. In Generation R, none of the six SNPs were associated with asthma. Fig. 1 shows the Forest plots for the weighted per‐allele associations of the six SNPs with asthma. For all six SNPs, the pooled effect estimates confirmed significant associations with asthma.

### Gene main effects in other European asthma studies

Figs S2–S6 online show Forest plots for the meta‐analysis of the associations between *TRPA1* and childhood‐onset asthma across GABRIEL studies, for five of the six SNPs most significantly associated with asthma in ALSPAC (rs920829 was not genotyped in GABRIEL; it was imputed in ALSPAC, but it is in strong LD with rs3735945). The plots compare associations with current doctor‐diagnosed asthma in ALSPAC and PIAMA versus associations with doctor‐diagnosed asthma ‘ever’ (of childhood‐onset) across other GABRIEL studies, with three studies which were exclusively of children separated from remaining studies. The pooled effect estimates do not confirm associations with asthma ‘ever’. Furthermore, there was evidence of substantial heterogeneity in the effect estimates for the three childhood GABRIEL studies.

### Paracetamol analyses in ALSPAC

For the 13 SNPs associated with asthma (p < 0.05), we stratified the per‐allele associations by early and late gestation paracetamol exposure. Associations were similar in exposed and unexposed children for the six SNPs most significantly associated with asthma overall (Table 3) and for the remaining 7 SNPs (data not shown). For the three SNPs associated with IgE concentration (p < 0.05), we similarly stratified the per‐allele associations by prenatal paracetamol exposure (Table 4). *TRPA1* was associated with IgE concentration amongst children who were exposed, especially in later gestation, but not amongst non‐exposed children (p‐interaction 0.02 for rs959974 and rs1384001, and 0.06 for rs4738202).

**Table 3:** Per‐allele associations between the six most significantly associated *TRPA1* SNPs and current doctor‐diagnosed asthma, stratified by prenatal paracetamol exposure during early and late gestation in ALSPAC

SNP	N	Paracetamol early in pregnancy OR (95% C.I.)	p‐value	N	Paracetamol later in pregnancy OR (95% C.I.)	p‐value
rs959974						
Exposed	2734	1.29 (1.10–1.50)	0.002	2118	1.26 (1.06–1.50)	0.008
Unexposed	2338	1.37 (1.12–1.66)	0.002	2889	1.31 (1.10–1.56)	0.002
		p‐interaction	0.639		p‐interaction	0.765
rs1384001						
Exposed	2734	1.29 (1.10–1.50)	0.002	2118	1.26 (1.06–1.50)	0.008
Unexposed	2338	1.37 (1.12–1.66)	0.002	2889	1.31 (1.10–1.56)	0.002
		p‐interaction	0.643		p‐interaction	0.760
rs4738202						
Exposed	2734	1.22 (1.03–1.45)	0.024	2118	1.23 (1.02–1.49)	0.031
Unexposed	2338	1.24 (1.00–1.54)	0.049	2889	1.18 (0.97–1.43)	0.090
		p‐interaction	0.910		p‐interaction	0.753
rs7010969						
Exposed	2734	1.25 (1.07–1.47)	0.006	2118	1.25 (1.05–1.50)	0.012
Unexposed	2338	1.34 (1.09–1.64)	0.005	2889	1.31 (1.09–1.57)	0.003
		p‐interaction	0.621		p‐interaction	0.738
rs3735945						
Exposed	2734	1.15 (0.91–1.44)	0.242	2118	1.31 (1.02–1.68)	0.036
Unexposed	2338	1.59 (1.20–2.09)	0.001	2889	1.24 (0.96–1.60)	0.096
		p‐interaction	0.076		p‐interaction	0.755
rs920829						
Exposed	2732	1.15 (0.91–1.44)	0.238	2114	1.30 (1.01–1.67)	0.041
Unexposed	2335	1.57 (1.19–2.08)	0.001	2888	1.24 (0.96–1.61)	0.093
		p‐interaction	0.086		p‐interaction	0.809

**Table 4:** Per‐allele associations between the three most significantly associated *TRPA1* SNPs and total IgE concentration, stratified by prenatal paracetamol exposure during early and late gestation in ALSPAC

SNP	N	Paracetamol early in pregnancy GMR^a^ (95% C.I.)	p‐value	N	Paracetamol later in pregnancy GMR^a^ (95% C.I.)	p‐value
rs959974						
Exposed	2066	1.12 (1.01–1.24)	0.037	1587	1.22 (1.08–1.37)	0.001
Unexposed	1719	1.05 (0.94–1.17)	0.408	2149	1.01 (0.91–1.12)	0.849
		p‐interaction	0.414		p‐interaction	0.017
rs1384001						
Exposed	2066	1.12 (1.01–1.24)	0.037	1587	1.22 (1.08–1.37)	0.001
Unexposed	1719	1.05 (0.94–1.17)	0.402	2149	1.01 (0.91–1.12)	0.849
		p‐interaction	0.418		p‐interaction	0.016
rs4738202						
Exposed	2066	1.16 (1.03–1.29)	0.011	1587	1.21 (1.06–1.37)	0.003
Unexposed	1719	1.02 (0.91–1.15)	0.714	2149	1.03 (0.92–1.15)	0.585
		p‐interaction	0.145		p‐interaction	0.062

a Geometric Mean Ratio.

## Discussion

We found strong evidence for an association between *TRPA1* polymorphisms and asthma in children at 7–8 years of age in the population‐based ALSPAC birth cohort. Of the six SNPs most significantly associated with asthma in ALSPAC, three showed some evidence of association (and larger effect estimates) with a similar asthma phenotype in the PIAMA birth cohort, whilst none of the six SNPs were associated with asthma at 6 years in Generation R. However, both PIAMA and Generation R were considerably smaller and had a lower prevalence of current asthma, than ALSPAC, and hence lacked statistical power to replicate findings individually. When we meta‐analysed across all three birth cohorts, the pooled effect estimates confirmed associations with asthma overall. Given the *a priori* selection of SNPs, the level of statistical significance for the ‘top hits’ in the ALSPAC discovery data set, and supportive evidence in PIAMA and following meta‐analysis across all three cohorts, we believe these results may represent a causal influence of the *TRPA1* gene on the risk of active childhood asthma. Other genes in the vicinity of *TRPA1* are unlikely to explain our findings as there is little apparent LD extending between *TRPA1* and other nearby genes (1000 Genomes Phase 1 CEU (www.1000genomes.org)). To our knowledge, these findings are novel and suggest that TRPA1 may play a role in the development of childhood asthma. Whilst a recent study reported correlations between two *TRPA1* polymorphisms and asthma control in children with asthma 27, it was underpowered and statistical evidence was weak.

### Importance of asthma phenotype

There is likely to be genetic heterogeneity of asthma phenotypes in childhood 28, as demonstrated for adult asthma phenotypes 29. This may partly explain why *TRPA1* was not associated with asthma in the other European studies. A limitation of the GABRIEL asthma GWAS was that the asthma ‘ever’ phenotype was not directly comparable to the ‘current’ asthma phenotype used in ALSPAC, PIAMA and Generation R; a doctor diagnosis of asthma ‘ever’ is likely to comprise many different phenotypes or endotypes which, when analysed together, may lead to dilution of effects of genetic variants 30. For example, in children, ‘asthma ever’ may capture early transient childhood wheezing. We confirmed that the effect estimates for the association between *TRPA1* and asthma were smaller in ALSPAC, and especially in PIAMA, when we analysed ‘ever’ asthma rather than ‘current’ asthma in these cohorts. Other possible reasons for the lack of association across the other European studies include differences in how cases were selected, which may have contributed to heterogeneity of the asthma phenotype; unreliability of recall of childhood‐onset asthma amongst the adult studies in GABRIEL; and variation in the prevalence of environmental exposures that interact with the gene across different European populations 31.

### Mechanisms

Given that reactive oxygen species are thought to play an important role in the pathogenesis of airways disease 32, and the TRPA1 receptor is an important oxidant sensor expressed on sensory neurons innervating the airways 2, it seems plausible that TRPA1 may play a critical role in asthma pathogenesis. Activation of TRPA1 can, through release of neuropeptides, promote neurogenic airway inflammation 3, 5. Conversely, in murine models of airway inflammation induced by allergen, cigarette smoke and paracetamol, deletion or antagonism of TRPA1 has been shown to reduce airway inflammation and hyper‐reactivity 6, 10, 33. However, as neurogenic inflammation has not been demonstrated in human asthma, there are two other mechanisms to consider. First, TRPA1 may also influence airway inflammation non‐neuronally, as confirmed in animals 34, and recent *in vitro* studies have shown that *TRPA1* is functionally expressed in human lung, including pulmonary epithelial cells 34, 35, smooth muscle cells 34 and lung fibroblasts 35. Second, a neuronal reflex mechanism may be involved, as suggested by experiments in rodents 36.

The lack of modification of the association between *TRPA1* and asthma by prenatal paracetamol exposure suggests that, even if foetal TRPA1 is activated by exposure to the metabolite of paracetamol 10 *in utero*, this mechanism is unlikely to explain the association between prenatal paracetamol and asthma. The apparent interaction we observed between prenatal paracetamol exposure and *TRPA1* genotype on IgE concentration is intriguing, but may be a chance finding and we cannot offer a mechanistic explanation. We speculate that other prenatal and post‐natal oxidant exposures may be more important than paracetamol as activators of TRPA1, thus contributing to the association we have found between *TRPA1* genotype and childhood asthma.

## Conclusions and future work

Our findings suggest, for the first time, that TRPA1 may play a role in the development of childhood asthma. In terms of therapeutic implications, these data lend further support to the proposition that TRPA1 antagonists may have promising potential in asthma 4. It is important that our findings are further replicated in adequately powered studies with comparable asthma phenotypes, and we plan to explore interactions between *TRPA1* and other oxidant exposures such as tobacco smoke and air pollution on childhood respiratory outcomes.

## Funding

The UK Medical Research Council, the Wellcome Trust (Grant ref: 102215/2/13/2) and the University of Bristol provide core support for ALSPAC. The PIAMA study is supported by grants from the Dutch Lung Foundation (grant numbers 3.4.01.26, 3.2.06.022, and 3.2.09.081JU), ZonMw (the Netherlands Organization for Health Research and Development), the Netherlands Ministry of Spatial Planning, Housing and the Environment, the Netherlands Ministry of Health, Welfare and Sport. Genomewide genotyping in PIAMA was supported by BBMRI‐NL (CP29) and the European Commission (Gabriel study, contract number 018996). FND is supported by a grant from the Ubbo Emmius Foundation. The Generation R Study is made possible by financial support from the Erasmus Medical Center (Rotterdam), the Erasmus University Rotterdam and the Netherlands Organization for Health Research and Development (ZonMw; 21000074). Dr Vincent Jaddoe received an additional grant from the Netherlands Organization for Health Research and Development (ZonMw‐VIDI) and a European Research Council Consolidator Grant (ERC‐2014‐CoG‐648916). Dr Liesbeth Duijts received funding from the Lung Foundation Netherlands (no 3.2.12.089; 2012).

## Supporting information


**Figure S1.** Linkage disequilibrium between 31 child *TRPA1* SNPs in ALSPAC using the Haploview program. Values of r^2^ (×100) are shown.

**Figure S2.** Forest plot showing meta‐analysis of the per‐allele association between *TRPA1* rs959974 and asthma ‘ever’ across GABRIEL studies*.

**Figure S3.** Forest plot showing meta‐analysis of the per‐allele association between *TRPA1* rs1384001 and asthma ‘ever’ across GABRIEL studies.

**Figure S4.** Forest plot showing meta‐analysis of the per‐allele association between *TRPA1* rs4738202 and asthma ‘ever’ across GABRIEL studies.

**Figure S5.** Forest plot showing meta‐analysis of the per‐allele association between *TRPA1* rs7010969 and asthma ‘ever’ across GABRIEL studies.

**Figure S6.** Forest plot showing meta‐analysis of the per‐allele association between *TRPA1* rs3735945 and asthma ‘ever’ across GABRIEL studies.

**Table S1.** Summary of *TRPA1* genotype data, including SNP position, minor allele frequency (MAF) and whether SNP was genotyped or imputed in white ALSPAC children.
**Table S2.** Per‐allele associations between child *TRPA1* SNPs and total IgE (log transformed) at 7.5 years in ALSPAC.

**Appendix S1.** Methods.
