# Supplementary figures and images for: TRPA1 gene polymorphisms and childhood asthma
Source: Pediatr Allergy Immunol. 2016 Dec 8;28(2):191–8. doi: 10.1111/pai.12673 (PMC5324656; doi:10.1111/pai.12673)

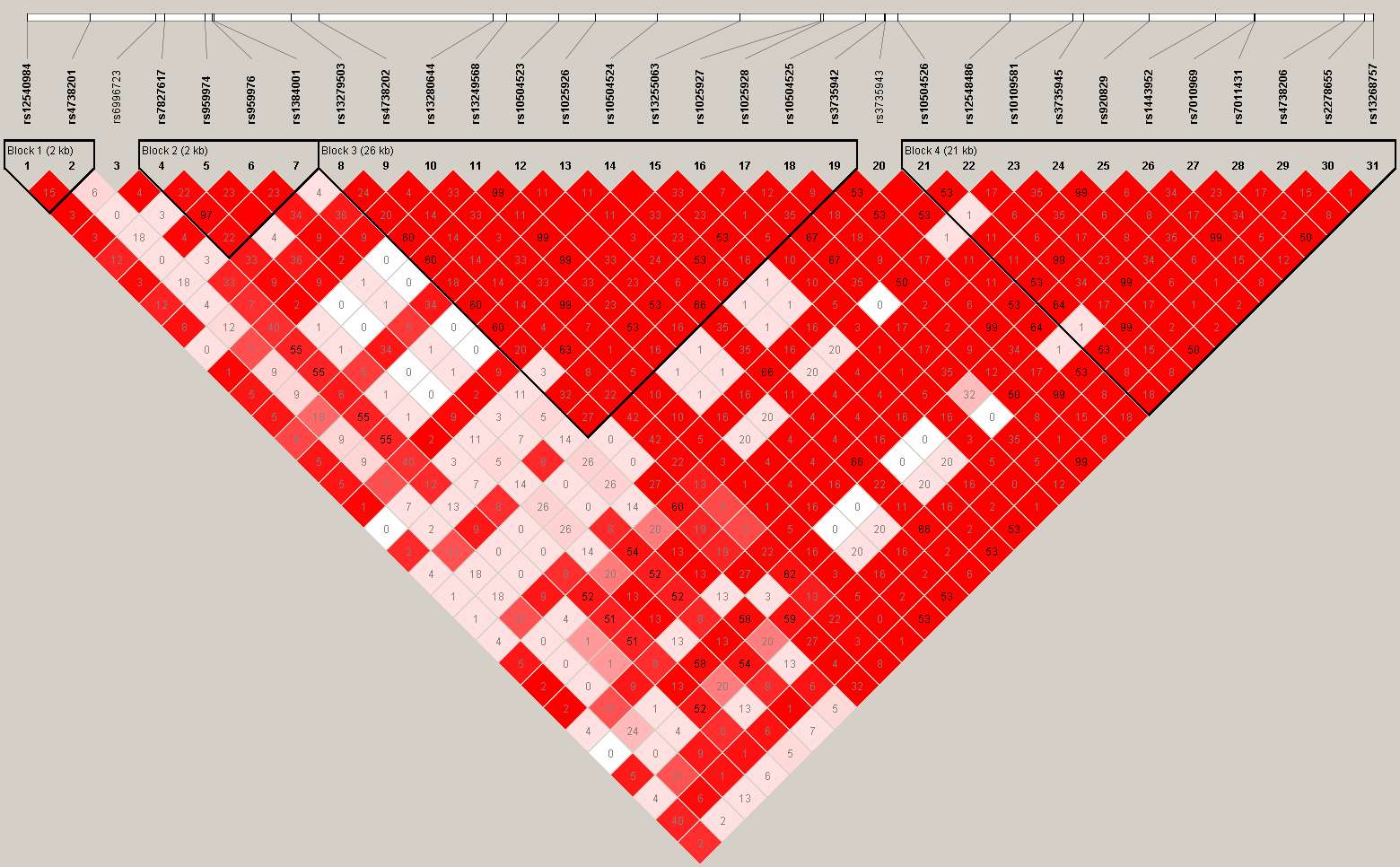

Supplement: Supplementary file 1 — Figure S1. Linkage disequilibrium between 31 child TRPA1 SNPs in ALSPAC using the Haploview program. Values of r2 (×100) are shown. [file PAI-28-191-s001.png]

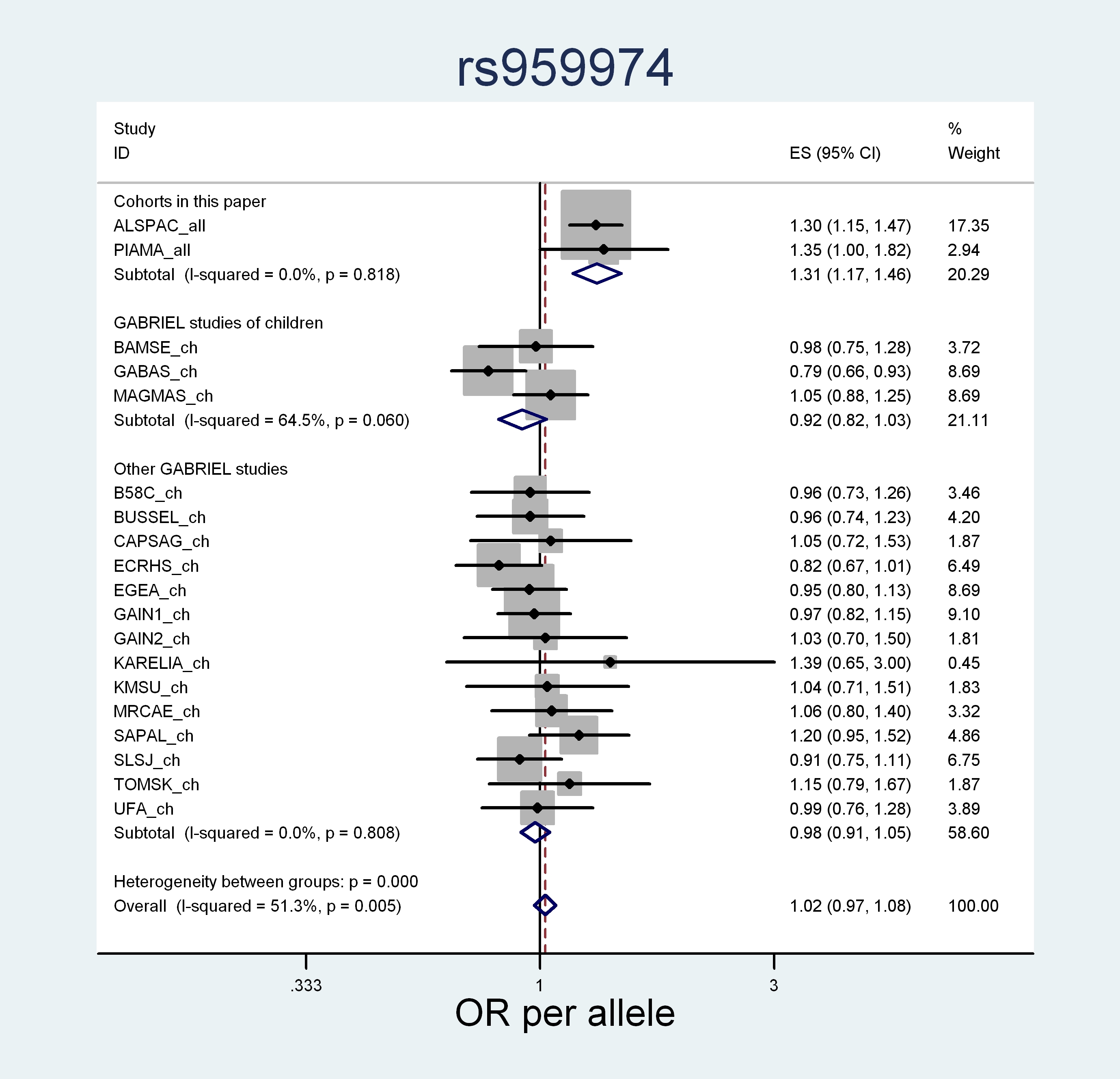

Supplement: Supplementary file 2 — Figure S2. Forest plot showing meta‐analysis of the per‐allele association between TRPA1 rs959974 and asthma ‘ever’ across GABRIEL studies*. [file PAI-28-191-s002.jpeg]

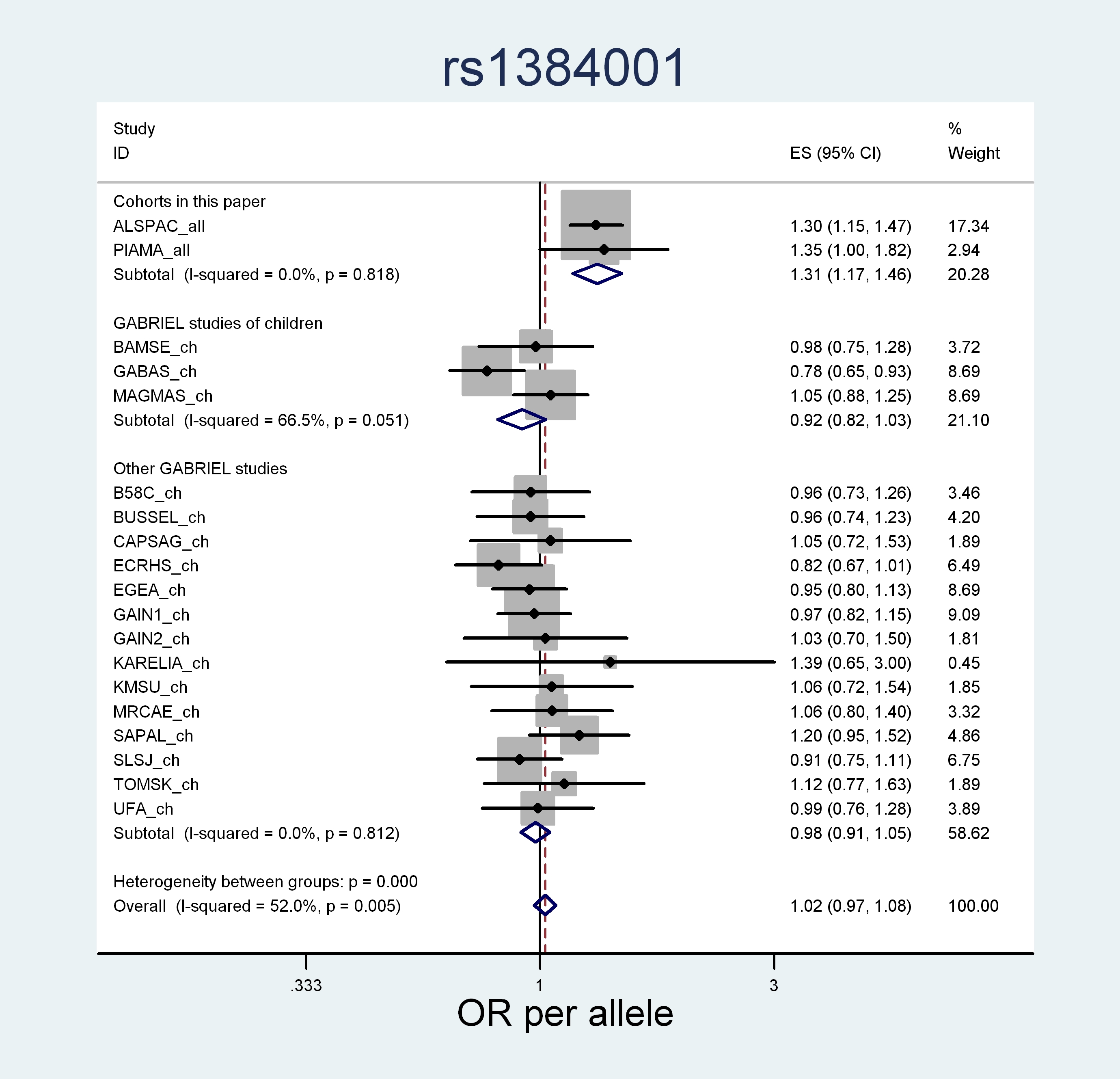

Supplement: Supplementary file 3 — Figure S3. Forest plot showing meta‐analysis of the per‐allele association between TRPA1 rs1384001 and asthma ‘ever’ across GABRIEL studies. [file PAI-28-191-s003.jpeg]

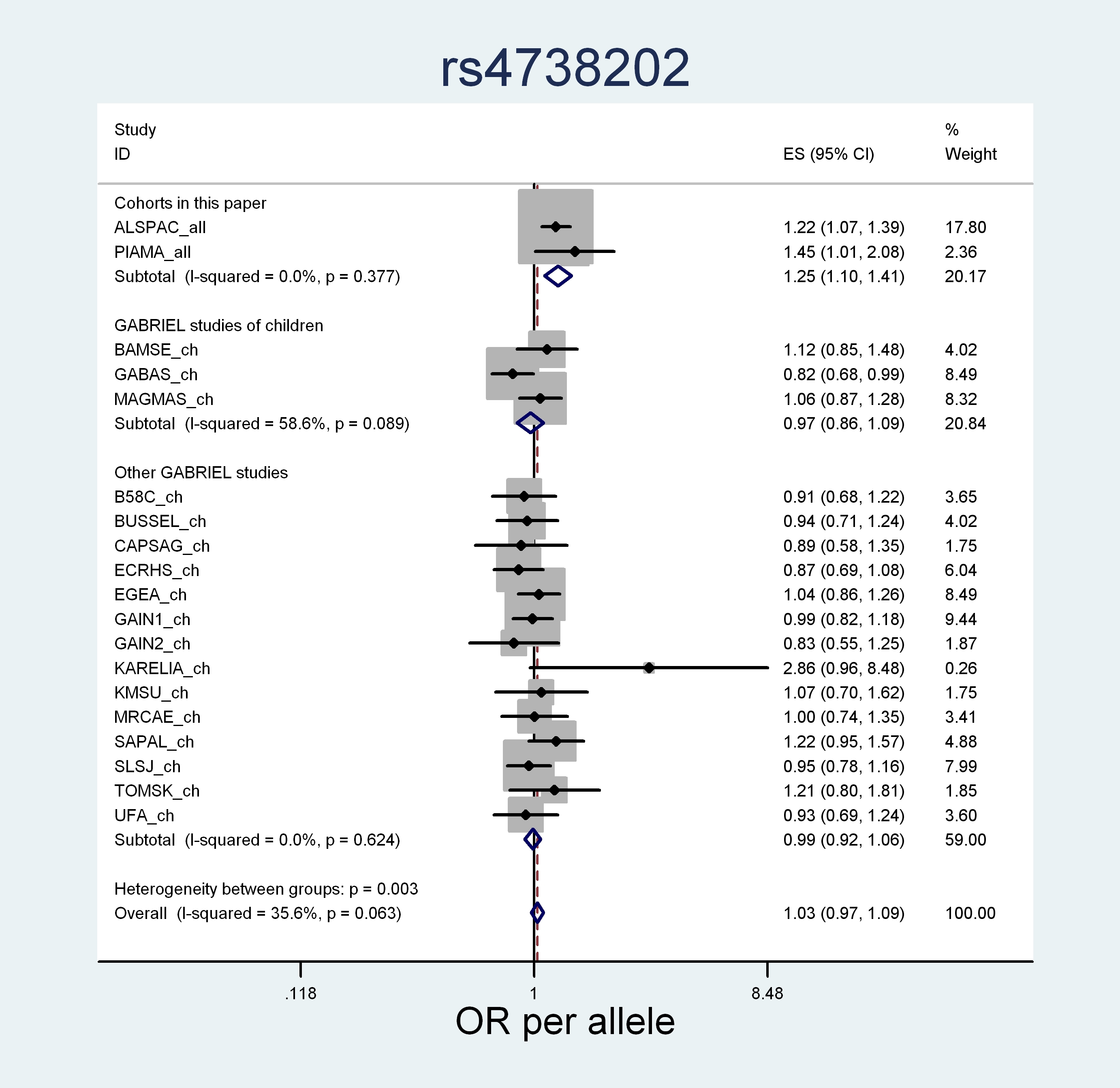

Supplement: Supplementary file 4 — Figure S4. Forest plot showing meta‐analysis of the per‐allele association between TRPA1 rs4738202 and asthma ‘ever’ across GABRIEL studies. [file PAI-28-191-s004.jpeg]

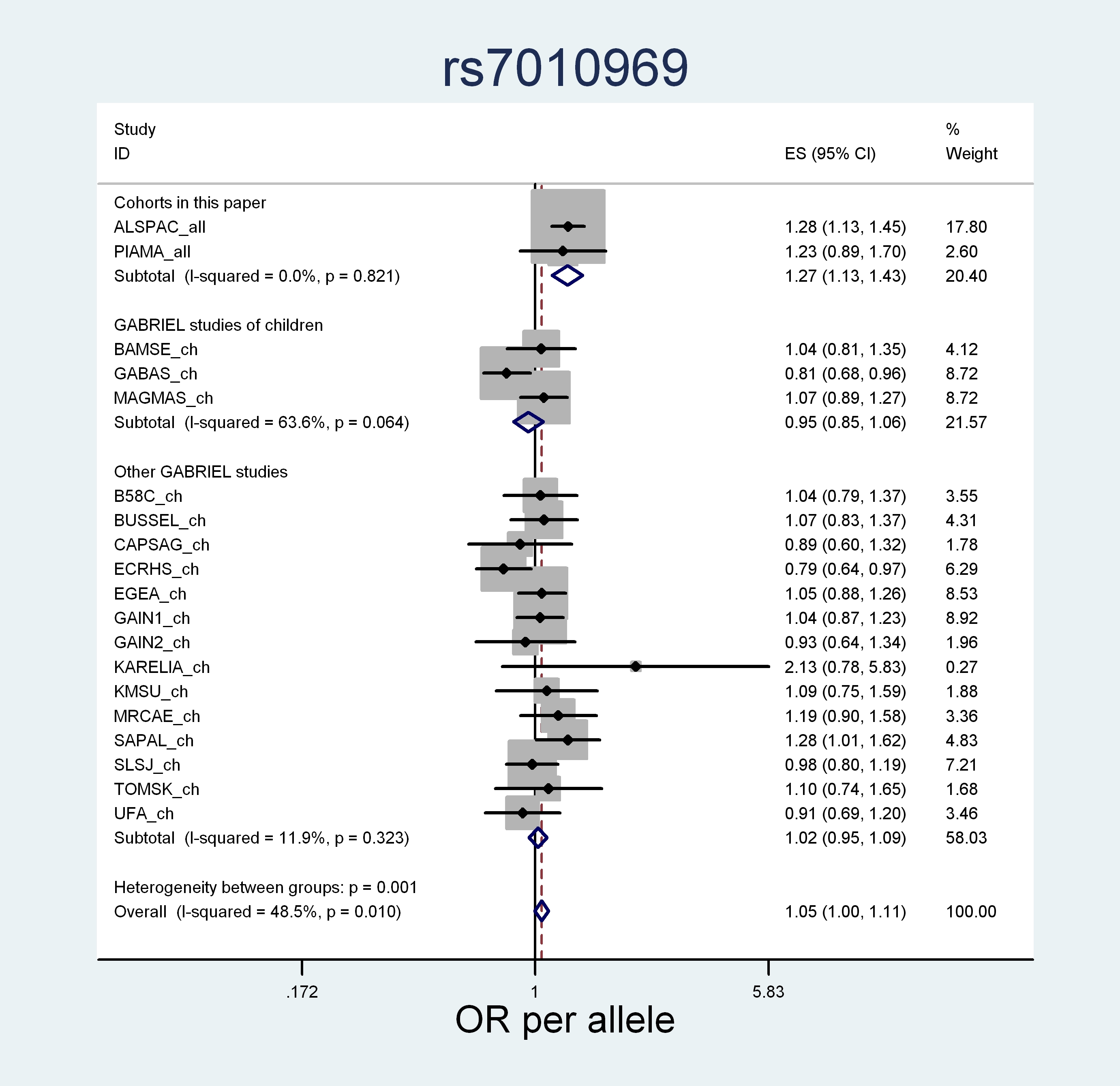

Supplement: Supplementary file 5 — Figure S5. Forest plot showing meta‐analysis of the per‐allele association between TRPA1 rs7010969 and asthma ‘ever’ across GABRIEL studies. [file PAI-28-191-s005.jpeg]

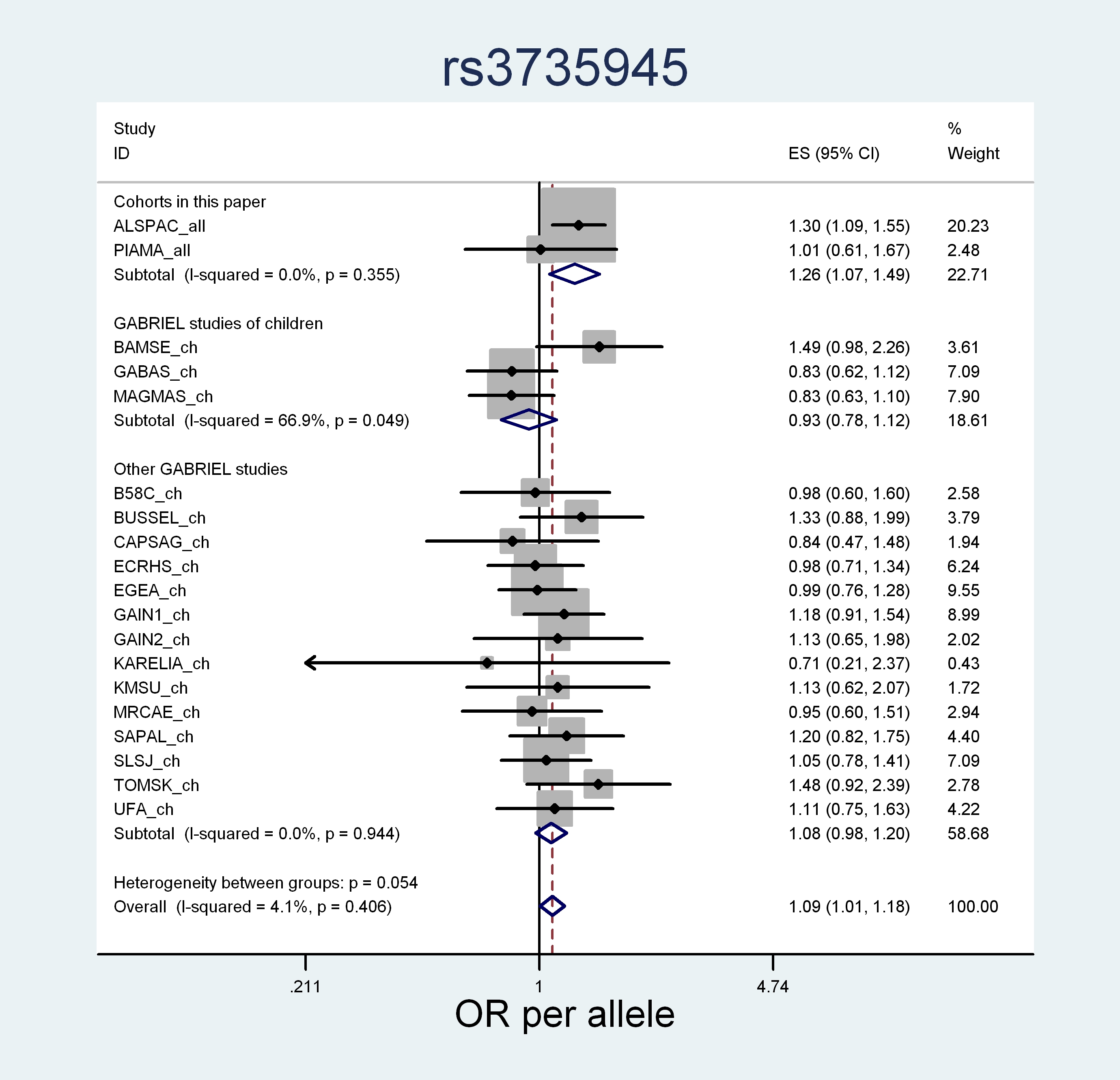

Supplement: Supplementary file 6 — Figure S6. Forest plot showing meta‐analysis of the per‐allele association between TRPA1 rs3735945 and asthma ‘ever’ across GABRIEL studies. [file PAI-28-191-s006.jpeg]
