# Supplementary material for: TRPA1 gene polymorphisms and childhood asthma
Source: Pediatr Allergy Immunol. 2016 Dec 8;28(2):191–8. doi: 10.1111/pai.12673 (PMC5324656; doi:10.1111/pai.12673)
Supplement: Supplementary file 7 — Table S1. Summary of TRPA1 genotype data, including SNP position, minor allele frequency (MAF) and whether SNP was genotyped or imputed in white ALSPAC children. Table S2. Per‐allele associations between child TRPA1 SNPs and total IgE (log transformed) at 7.5 years in ALSPAC. [file PAI-28-191-s007.docx]

Table S1: Summary of *TRPA1* genotype data, including SNP position, minor allele frequency (MAF) and whether SNP was genotyped or imputed in white ALSPAC children

| SNP | Position | Allele* | MAF | SNP genotyped | N missing replaced with imputed | SNP imputed | N |
| --- | --- | --- | --- | --- | --- | --- | --- |
| rs12540984 | 72927920 | A/G | 0.1425 | - |  | 5,034 A/A 1,683 A/G 143 G/G | 6,860 |
| rs4738201 | 72930711 | A/G | 0.4859 | 1,621 A/A  3,419 A/G  1,860 G/G | 41 | - | 6,900 |
| rs6996723 | 72933632 | C/T | 0.1769 | 4,664 C/C 2,024 T/C 213 T/T | 0 | - | 6,901 |
| rs7827617 | 72934032 | A/G | 0.1711 | 4,758 A/A 1,937 A/G 206 G/G | 25 | - | 6,901 |
| rs959974 | 72935839 | G/T | 0.4708 | 1,920 G/G 3,476 T/G 1,505 T/T | 1 | - | 6,901 |
| rs959976 | 72936145 | T/C | 0.1717 | 4,751 T/T 1,942 C/T  208 C/C | 4 | - | 6,901 |
| rs1384001 | 72936237 | C/A | 0.4708 | 1,919 C/C 3,477 A/C  1,505 A/A | 1 | - | 6,901 |
| rs13279503 | 72939626 | G/C | 0.3545 | - | - | 2,854 G/G 3,168 C/G  848 C/C | 6,870 |
| rs4738202 | 72940861 | A/G | 0.3105 | 662 A/A  2,985 A/G  3,254 G/G | 16 | - | 6,901 |
| rs13280644 | 72948588 | C/T | 0.0982 | 5,608 C/C  1,227 T/C 66 T/T | 0 | - | 6,901 |
| rs13249568 | 72949209 | T/C | 0.2495 | 3,866 T/T 2,620 C/T  415 C/C | 8 | - | 6,901 |
| rs10504523 | 72951490 | G/A | 0.2493 | 3,868 G/G 2,619 A/G  414 A/A | 3 | - | 6,901 |
| rs1025926 | 72953158 | C/T | 0.251 | 3,856 C/C 2,611 T/C 434 T/T | 51 | - | 6,901 |
| rs10504524 | 72955891 | G/T | 0.2493 | - | - | 3,868 G/G 2,618 G/T 415 T/T | 6,901 |
| rs13255063 | 72959535 | T/A | 0.2493 | - | - | 3,867 T/T 2,618 A/T 415 A/A | 6,900 |
| rs1025927 | 72963135 | A/G | 0.0984 | - | - | 5,601 A/A 1,231 A/G 66 G/G | 6,898 |
| rs1025928 | 72963258 | T/C | 0.4156 | 1,188 T/T 3,362 T/C  2,351 C/C | 0 | - | 6,901 |
| rs10504525 | 72965123 | C/T | 0.1508 | 4,966 C/C 1,788 T/C 147 T/T | 14 | - | 6,901 |
| rs3735942 | 72965973 | G/A | 0.335 | - | - | 3,066 G/G 3,058 A/G 777 A/A | 6,901 |
| rs3735943 | 72966002 | G/A | 0.4858 | 1,838 G/G 3,431 A/G 1,632 A/A | 2 | - | 6,901 |
| rs10504526 | 72966552 | A/G | 0.4858 | 1,837 A/A 3,432 G/A 1,632 G/G | 3 | - | 6,901 |
| rs12548486 | 72971527 | C/T | 0.3351 | - | - | 3,061 C/C 3,059 C/T 777 T/T | 6,897 |
| rs10109581 | 72974329 | G/T | 0.2599 | 3,767 G/G 2,669 T/G 465 T/T | 1 | - | 6,901 |
| rs3735945 | 72974806 | C/T | 0.1091 | 5,478 C/C 1,332 T/C 91 T/T | 1 | - | 6,901 |
| rs920829 | 72977703 | C/T | 0.1091 | - | - | 5,477 C/C 1,328 C/T 91 T/T | 6,896 |
| rs1443952 | 72980652 | C/T | 0.3351 | 3,064 C/C 3,060 T/C 777 T/T | 8 | - | 6,901 |
| rs7010969 | 72982365 | A/C | 0.405 | 1,139 A/A 3,307 A/C  2,455 C/C | 36 | - | 6,901 |
| rs7011431 | 72982398 | G/A | 0.26 | 3,769 G/G 2,663 A/G 469 A/A | 0 | - | 6,901 |
| rs4738206 | 72986348 | T/G | 0.3343 | 3,076 T/T 3,054 G/T 771 G/G | 1 | - | 6,901 |
| rs2278655 | 72987277 | C/T | 0.071 | - | - | 5,889 C/C 836 C/T 32 T/T | 6,757 |
| rs13268757 | 72987638 | G/A | 0.1492 | - | - | 4,962 G/G 1,742 A/G 136 A/A | 6,840 |

*Allele order as per Ensembl database (<http://www.ensembl.org/index.html>)

Table S2: Per-allele associations between child *TRPA1* SNPs and total IgE (log transformed) at 7.5 years in ALSPAC

| **SNP** | **Position** | **Ln-IgE levels** | | |
| --- | --- | --- | --- | --- |
|  |  | **N** | **OR (95% CI)** | **P value** |
| rs12540984 | 72927920 | 3808 | 0.93 (0.84-1.04) | 0.210 |
| rs4738201 | 72930711 | 3834 | 1.05 (0.97-1.13) | 0.203 |
| rs6996723 | 72933632 | 3834 | 1.06 (0.96-1.17) | 0.262 |
| rs7827617 | 72934032 | 3834 | 1.03 (0.93-1.14) | 0.574 |
| rs959974 | 72935839 | 3834 | 1.08 (1.00-1.7) | 0.043* |
| rs959976 | 72936145 | 3834 | 1.03 (0.94-1.14) | 0.533 |
| rs1384001 | 72936237 | 3834 | 1.08 (1.00-1.17) | 0.042* |
| rs13279503 | 72939626 | 3822 | 1.06 (0.98-1.15) | 0.119 |
| rs4738202 | 72940861 | 3834 | 1.09 (1.01-1.19) | 0.031* |
| rs13280644 | 72948588 | 3834 | 1.06 (0.93-1.20) | 0.391 |
| rs13249568 | 72949209 | 3834 | 1.07 (0.98-1.17) | 0.140 |
| rs10504523 | 72951490 | 3834 | 1.07 (0.98-1.17) | 0.139 |
| rs1025926 | 72953158 | 3834 | 1.04 (0.95-1.14) | 0.361 |
| rs10504524 | 72955891 | 3834 | 1.07 (0.98-1.17) | 0.139 |
| rs13255063 | 72959535 | 3834 | 1.07 (0.98-1.17) | 0.139 |
| rs1025927 | 72963135 | 3832 | 1.06 (0.93-1.20) | 0.373 |
| rs1025928 | 72963258 | 3834 | 0.94 (0.87-1.02) | 0.115 |
| rs10504525 | 72965123 | 3834 | 1.05 (0.95-1.17) | 0.317 |
| rs3735942 | 72965973 | 3834 | 1.01 (0.93-1.10) | 0.767 |
| rs3735943 | 72966002 | 3834 | 0.96 (0.89-1.04) | 0.319 |
| rs10504526 | 72966552 | 3834 | 1.04 (0.96-1.12) | 0.308 |
| rs12548486 | 72971527 | 3832 | 1.01 (0.93-1.10) | 0.768 |
| rs10109581 | 72974329 | 3834 | 1.05 (0.97-1.15) | 0.218 |
| rs3735945 | 72974806 | 3834 | 1.04 (0.92-1.17) | 0.576 |
| rs920829 | 72977703 | 3831 | 1.04 (0.92-1.17) | 0.530 |
| rs1443952 | 72980652 | 3834 | 1.02 (0.94-1.10) | 0.694 |
| rs7010969 | 72982365 | 3834 | 1.06 (0.98-1.14) | 0.166 |
| rs7011431 | 72982398 | 3834 | 1.05 (0.97-1.14) | 0.247 |
| rs4738206 | 72986348 | 3834 | 1.01 (0.93-1.09) | 0.795 |
| rs2278655 | 72987277 | 3751 | 0.91 (0.78-1.06) | 0.205 |
| rs13268757 | 72987638 | 3803 | 1.06 (0.95-1.18) | 0.288 |

*p-value<0.05
