# Supplementary material for: TRPA1 gene polymorphisms and childhood asthma
Source: Pediatr Allergy Immunol. 2016 Dec 8;28(2):191–8. doi: 10.1111/pai.12673 (PMC5324656; doi:10.1111/pai.12673)
Supplement: Supplementary file 8 — Appendix S1. Methods. [file PAI-28-191-s008.docx]

**Online Supplement**

**Methods**

ALSPAC GWAS data generation and imputation methodology

In order to obtain the most robust GWAS data, individuals were excluded from further analysis on the basis of having: incorrect gender assignments; minimal or excessive heterozygosity (<0.320 and >0.345 for the Sanger data and <0.310 and >0.330 for the LabCorp data); disproportionate levels of individual missingness (>3%); evidence of cryptic relatedness (>10% Identity by Descent, IBD); and being of non-European ancestry (as detected by a multidimensional scaling analysis seeded with HapMap 2 individuals) in order to reduce the possibility of confounding by population substructure. EIGENSTRAT principal components analysis was used to generate the top 100 principal components after the removal of known regions of long linkage disequilibrium in the data^1;2^. This revealed no additional obvious population stratification and genome-wide analyses with other phenotypes indicate a low lambda. SNPs with a minor allele frequency of <1% and call rate of <95% were removed. Furthermore, only SNPs which passed an exact test of Hardy–Weinberg equilibrium (P>5×10-7) were considered for analysis.

Known autosomal variants were imputed with MACH 1.0.16 Markov Chain Haplotyping software^3;4^, using Centre d’etude du polymorphisme humain (CEPH) individuals from phase 2 of the HapMap^5^ project (HG18) as a reference set (release 22). For each imputed SNP of interest, dosages were estimated based on probabilities calculated by the MACH algorithm (where 0 is the first homozygote, 1 is a perfect heterozygote, and 2 is the other homozygote); imputed genotypes were set to missing if the dosage was >0.3 either side of the integer dose.

PIAMA questionnaire data, genotyping and imputation methodology, and ethics

Questionnaires for parental completion, partly based on the International Study of Asthma and Allergies in Childhood core questionnaires, were sent to parents when the child was 8 years old. If parents confirmed that the child had been diagnosed with asthma by a doctor (ever), and had had asthma and/or one or more attacks of wheeze in the last 12 months, they were regarded as having current doctor-diagnosed asthma.

DNA was extracted from blood or buccal swabs at the age of 4 or 8 years. Children were genotyped on 3 different platforms. DNA of 1377 children was genotyped on the Illumina Omni Express Exome Chip and DNA of 288 children was genotyped with the Omni Express chip, both at the Genomics Facility of the University Medical Center Groningen. DNA of 404 children was genotyped at the Centre National de Genotypage (CNG, Evry, France) as part of the GABRIEL consortium^6^. SNPs were harmonized by base pair position annotated to genome build 37, name and annotation of strand for each platform. Discordant or duplicate SNPs or SNPs that showed large differences in allele frequencies (> 15 %) were removed. After quality control, a total of 1968 individuals remained and imputation was performed per platform using IMPUTE 2,0 against the reference data set of the CEU panel of the 1000 Genomes project (version March 2012). SNPs of high quality (info-score IMPUTE ≥ 0.7) were merged into one dataset using GTOOL and used for further analysis. Dosages of imputed SNPs were predicted based on the following assumptions; 0 is the first homozygote, 0.5 is a heterozygote and 1 is the other homozygote. The info scores of the imputed SNPs were all ≥0.99 which provided reliable dosages estimates. Analyses of SNPs most significantly associated with asthma in ALSPAC were performed using SNPtest v2.4.1 and IBM SPSS Statistics for Windows (Version 22.0, Armonk, NY).

The Medical Ethical Committees of the participating institutes approved the study, and all participants gave written informed consent.

Generation R questionnaire data, genotyping and imputation technology, and ethics

Information about wheezing and asthma was collected by a parental questionnaire at age 6 years^7^. Response rate for this questionnaire was 68%. Asthma was assessed with the question ‘Was your child ever diagnosed with asthma by a doctor? [no; yes]’. Wheezing was assessed with the question ‘Did your child ever suffer from wheezing in the last 12 months? [never, 1-3 times, >4 times]’. If parents confirmed that the child had been diagnosed with asthma by a doctor and had >=1 wheezing episode in the last 12 months, they were regarded as having current doctor-diagnosed asthma [no; yes].

Cord blood samples including DNA were collected at birth. Samples were genotyped using Illumina Infinium II HumanHap610 Quad Arrays following standard manufacturer's protocols. Intensity files were analyzed using the Beadstudio Genotyping Module software v.3.2.32 and genotype calling based on default cluster files. Any sample displaying call rates below 97.5%, excess of autosomal heterozygosity (F<mean-4SD), and mismatch between called and phenotypic gender were excluded. In addition, individuals identified as genetic outliers by the IBS clustering analysis (> 3 standard deviations away from the HapMap CEU population mean) were excluded from the analysis. Genotypes were imputed for all polymorphic SNPs from phased haplotypes in autosomal chromosomes using the 1000 Genomes GIANTv3 panel. Analyses of single nucleotide polymorphisms (SNPs) most significantly associated with asthma in ALSPAC were performed using IBM SPSS Statistics for Windows (Version 21.0, Chicago, IL).

The study protocol was approved by the Medical Ethical Committee of the Erasmus Medical Centre, Rotterdam (MEC 217.595/2002/20). Written informed consent was obtained from parents of all participants.

**References**

(1) Price AL, Patterson NJ, Plenge RM, Weinblatt ME, Shadick NA, Reich D. Principal components analysis corrects for stratification in genome-wide association studies. *Nat Genet* 2006; 38:904-909.

(2) Price AL, Weale ME, Patterson N, Myers SR, Need AC, Shianna KV et al. Long-Range LD Can Confound Genome Scans in Admixed Populations. Am *J Hum Genet* 2008; 83:132-135.

(3) Li Y, Willer C, Sanna S, Abecasis G. Genotype Imputation. *Annu Rev Genom Human Genet* 2009; 10(1):387-406.

(4) Li Y, Willer CJ, Ding J, Scheet P, Abecasis GR. MaCH: using sequence and genotype data to estimate haplotypes and unobserved genotypes. *Genet Epidemiol* 2010; 34:816-834.

(5) The International HapMap Project. *Nature* 2003; 426:789-796.

(6) Moffatt MF, Gut IG, Demenais F, Strachan DP, Bouzigon E, Heath S et al. A Large-Scale, Consortium-Based Genomewide Association Study of Asthma. *N Engl J Med* 2010; 363:1211-1221.

(7) Jaddoe VWV, van Duijn CM, van der Heijden AJ, Mackenbach JP, Moll HtA, Steegers EAP et al. The Generation R Study: design and cohort update 2010. *Eur J Epidemiol* 2010; 25:823-841.

**Figure E1: Linkage disequilibrium between 31 child *TRPA1* SNPs in ALSPAC using the Haploview program. Values of r^2^ (x100) are shown.**

**Figure E2: Forest plot showing meta-analysis of the per-allele association between *TRPA1* rs959974 and current asthma in ALSPAC and PIAMA, and asthma ‘ever’ across other GABRIEL studies**

**Figure E3: Forest plot showing meta-analysis of the per-allele association between *TRPA1* rs1384001 and current asthma in ALSPAC and PIAMA, and asthma ‘ever’ across other GABRIEL studies**

**Figure E4: Forest plot showing meta-analysis of the per-allele association between *TRPA1* rs4738202 and current asthma in ALSPAC and PIAMA, and asthma ‘ever’ across other GABRIEL studies**

**Figure E5: Forest plot showing meta-analysis of the per-allele association between *TRPA1* rs7010969 and current asthma in ALSPAC and PIAMA, and asthma ‘ever’ across other GABRIEL studies**

**Figure E6: Forest plot showing meta-analysis of the per-allele association between *TRPA1* rs3735945 and current asthma in ALSPAC and PIAMA, and asthma ‘ever’ across other GABRIEL studies**

**Key for study ID** **in Figures** (_ch suffix denotes childhood onset asthma (ever), even if based on recall in adults; _all suffix denotes whole cohort analysis (current doctor-diagnosed asthma) as per Results in Table 2):

GABAS: GABRIEL Advanced Surveys (Germany)

MAGMAS: MAGICS (Multicentre Asthma Genetics in Childhood Study) and MAS (Multicentre Allergy Study) (Germany)

BAMSE: BAMSE cohort (Sweden)

EGEA: Genetics and Environment of Asthma (France)

TOMSK: Tomsk study (Russia); UFA: Ufa study (Russia)

ECRHS: European Community Respiratory Health Survey (Europe multicentre)

SAPAL: SAPALDIA (The Swiss study on Air Pollution and Lung Disease In Adults) (Switzerland)

KARELIA: Karelia Allergy Study (Finland)

KMSU: KMSU cohort (Russia)

MRCAE: MRCA and UKC (UK)

B58C: British 1958 Birth Cohort (UK)

BUSSEL: Busselton Health Study (Australia)

SLSJ: Saguenay-Lac-Saint-Jean Familial Collection (Quebec, Canada)

CAPSAG: Canadian Asthma Primary Prevention Study (CAPPS) and the Study of Asthma Genes and Environment (SAGE) (Canada)

GAIN1/GAIN2: Genetics of Asthma International Network (GAIN) (Multicentre)
